# Leveraging Leadership Development to Pre-Empt Leader Derailments

**DOI:** 10.3390/bs14121122

**Published:** 2024-11-23

**Authors:** Jay A. Conger

**Affiliations:** Psychology Department, Claremont McKenna College, Claremont, CA 91711, USA; jay.conger@cmc.edu; Tel.: +1-310-623-7036

**Keywords:** leader derailments, leadership failures, leadership development, leadership disorders

## Abstract

This article examines the role of leadership development interventions in pre-empting leader derailments. The research literature suggests that derailments are not only commonplace but associated with a range of significant costs, from financial, to mental health, to morale, to employee turnover, to missed opportunities. Given these costly consequences, this article seeks to answer the question: “Can leadership development—especially at early managerial-career stages and during transitions—play a significant role in mitigating leader derailments?” Research suggests that the majority of leadership failures occur—or are more visible—at senior organizational levels. This begs the question of whether development interventions earlier in a leader’s career might have pre-empted their later failure. What if the field of leadership development were to adopt a ‘preventative medicine’ model in which pre-empting derailments was the focus rather than ‘fixing’ leaders as they are derailing? Moreover, there appears to be an overreliance on coaching as the intervention of choice. What if the field were to leverage a broader range of development interventions beyond coaching to ensure a greater probability of minimizing derailments? Five interventions will be discussed which have the potential to pre-empt leadership failures, when deployed in unison.

## 1. Introduction

In 2024 alone, an estimated USD 81 billion was spent across a spectrum of leadership development interventions, from assessments to coaching to training. Most of these initiatives had a singular goal—instilling the core competencies or ‘right stuff’ associated with effective leadership. In contrast, it is rare to find leadership development initiatives focused on mitigating the ‘wrong stuff’—the factors that contribute to leadership failures. This bias towards the constructive or ‘light side’ dimensions of leadership has been reinforced by the popularity of positive organizational scholarship [1,2], inventories such as the Clifton Strengths, and the majority of leadership 360-feedback surveys, as well as developmental feedback emphasizing an individual’s strengths [3]. As a result, the field has paid limited attention to harmful competencies or ‘leader derailers’ [4,5]. As Pfeffer (2015) notes, there are few incentives to do otherwise [6]. The industry of leadership development has a ‘product’ to sell—a ‘feel-good prescription’ that promises higher performance and employee morale. The process of addressing leadership failures—a more complex and potentially intractable problem—is less amenable to productization and popular appeal.

The leadership development field appears to operate under the mistaken assumption that instilling constructive competences will somehow mitigate derailing behaviors. As Hogan, Hogan and Kaiser (2010) note, leader incompetence has been conceptualized as not possessing the characteristics of effective leadership or the ‘right stuff’ [7]. In reality, dysfunctional leader behavior is not the face of a coin, the other side of which is functional behavior. Leader failure is the product of undesirable characteristics or the ‘wrong stuff’—a different set of behaviors. Addressing these—often interpersonal—deficits requires interventions that are distinct from those associated with developing constructive leadership skills or the ‘right stuff’. While empirical research has advanced to provide reliable, evidence-based guidance for designing and delivering interventions to instill the ‘right stuff’ [8,9], research on interventions to mitigate leader derailments has not. This is a critical problem given the high and varied costs of derailments. In addition, there appears to be a heavy reliance on coaching as the primary intervention to ‘fix’ a derailing leader. This intervention can come with potential drawbacks [10]. Most significant among the drawbacks is the issue of timing. Coaching tends to be initiated after a manager has begun to visibly derail. Once identified to be derailing, the manager and their superior enter a ‘set up to fail’ syndrome [11]. This dynamic plays out as follows. As a manager’s performance slips to being unsatisfactory, the superior increases their attention towards and supervision of the derailing individual. Rather than improving the subordinate’s performance, the increased supervision has the reverse effect. Perceiving the boss’s lack of confidence in them, the subordinate often withdraws from his or her work and from their superior. As a result, the relationship spirals downward, as does performance [11]. Beyond this ‘set up to fail’ syndrome associated with their superiors, derailing managers may have harmed their working relationships with subordinates and peers. This is highly likely given the fact that derailments are often the product of flawed interpersonal tactics which work against the building and maintaining of trusting relationships [12]. While subordinates may experience these flaws more acutely [13], the same interpersonal shortcomings can weaken peer relationships. Research shows that the latter are essential to a manager’s overall performance [14]. Without positive working relationships, the derailing individual’s influence and performance further wanes. Complicating matters, derailing managers are typically characterized by low levels of self-awareness and self-regulation [7]. They tend to externalize the sources for their derailment. As a result, their response to coaching can be either defensive or manipulative—‘playing along’ or only making superficial changes to appear compliant in addressing the concerns of their superiors [10]. For these very reasons, coaching may fall far short in mitigating a leader’s derailment. ‘Preventative coaching’ occurring far earlier in a manager’s career may offer a better return on the development investment. But for various reasons described later in this article, superiors and organizations often overlook the early warning signs of leadership derailers, or the ‘wrong stuff’. As a result, they fail to take pre-emptive actions.

It is fair to ask what is the overall magnitude of the phenomenon of leaders who are perceived to be derailing? Hogan, Hogan and Kaiser (2010) cite twelve different studies, suggesting that the base rate of managerial failures ranges from 30% to 67%, with a mean of 47% and a median of 50% [7]. These estimates are reasonably consistent despite the fact that they come from distinctly different sources and samples. Examining CEO ousters alone, Stanford professor Larcker and his colleagues [15] examined nearly 1400 turnover events at Russell 3000 companies between 2017 and 2021. They found that 29% appeared to be involuntary or forced departures. For the general managerial population, Hogan, Hogan and Kaiser (2010) propose a base rate of fifty percent of managers being ineffective and possessing derailment potential [7]. Either way, the rates of leader derailments are remarkably high across hierarchical levels and sectors. Leader derailment is a not only costly but widespread phenomenon. Given the magnitude of the problem, it is surprising that the field of leadership development has devoted relatively little attention to addressing the issue.

From the perspective of leadership derailment, this article seeks to answer two questions: Why not devote a greater level of developmental resources to pre-empting the ‘wrong stuff’ earlier in a career? Why not move beyond an overreliance on ‘last minute’ coaching interventions to a ‘preventative medicine’ model deploying a suite of interventions? For example, to build greater self-awareness of one’s potential to derail, training initiatives could expose junior managers to common leader-derailers and disorders and their consequences. Assessments could be deployed to help individuals identify their susceptibility to derailers. Onboarding programs could provide newly promoted leaders with ‘real time’ feedback on the impact of their behaviors at the start of a new role along with corrective coaching and feedback. Later in this article, we will propose a suite of interventions with the potential to mitigate future derailments. 

For the purpose of this article, leadership derailment is defined as the process by which a leader who was previously seen as effective or identified as high-potential experiences a serious decline in efficacy and/or performance. These outcomes lead to diminished influence, career stagnation and organizational challenges such as morale or retention issues with subordinates or highly conflictual relations with peers or superiors. 

## 2. The Many Costs of Leader Derailments

It is important to ask whether there is a pressing need for the field of leadership development to prioritize addressing leader derailments. From the standpoint of its associated costs, the answer is a resounding ‘yes’. To start, leader derailment costs come in many forms—from financial costs, to missed organizational opportunities, to losses associated with employee morale and engagement. These costs in the United States alone are estimated to run in the tens of billions of dollars. The easiest ones to measure are those associated with executive and CEO-level failures. At the CEO level, failure comes with more dramatic costs (Conger and Nadler, 2004) [16]. If an executive recruiting firm is hired to find a replacement for the failed CEO, its fees can run to 25–33% of the first-year salary. In a matter of weeks, a floundering CEO can destroy a company’s market valuation that has taken a decade to build. Recent research suggests that the market value wiped out by CEO and C-suite failures in the S&P 1500 is close to USD 1 trillion a year [17]. Ousted CEOs rarely leave with empty pockets. A typical severance package provides the departing CEO of a Fortune 500 company with up to three times their annual salary plus a bonus, and extras can include compensation for life insurance, a USD 500,000 to USD 1 million annual payment for life, and office assistance for several years. An extreme example would be Adam Neumann, the ousted CEO of WeWork. Neumann departed with a USD 185 million noncompete agreement, a USD 106 million settlement payment, and USD 578 million for shares sold to the investor SoftBank. The forced departures of CEOs are often followed by shareholder class-action suits brought on by the plunging stock price, which presents another hazard. In 2023, USD 7.9 billion was paid out for these investor recoveries. Last but not least, the departing CEO’s organizational initiatives go into limbo during a transition crisis at the top. Important competitive advantages may be lost during this time.

While less visible, significant—though harder to quantify—costs are found below the executive levels of leadership. These are associated with losses in morale and mental and physical health experienced by the subordinates of derailed leaders [18]. Organizational climate surveys routinely show that about 75% of working adults report that their immediate supervisor is the most stressful aspect of their job [19]. As Hogan, Hogan and Kaiser (2010) point out, ineffective managers are a health hazard; they undermine the quality of life of many individuals and in turn impose medical costs on society [7]. Tepper (2000) and Skogstad, Einarsen, Torsheim, Aasland and Hetland (2007) have demonstrated how consequential these costs are empirically [20,21].

## 3. What We Have Learned from the Research on Leadership Derailment

What is surprising about the leader derailment research is the remarkable degree of convergence across findings. A central theme is that three interrelated factors combine to produce a leader’s downfall. Derailing leaders lack sufficient levels of self-awareness and self-regulation to navigate the impacts of their personality disorders. These disorders manifest themselves in dysfunctional behaviors that undermine their relationships with superiors, peers and/or subordinates. As a result, they are unable to meet performance expectations at a certain point in their careers, and in turn, derail. A brief history of the research on leadership derailment is highlighted below.

The pioneer of research on leader derailment was Jon Bentz (1967, 1990) [22,23], who undertook a 30-year study of failed managers at Sears, Roebuck and Company. While the derailed individuals he researched were described as bright and socially skilled, they failed for a variety of reasons. These included the following factors: (1) a lack of business skills, (2) a reactive and tactical disposition, (3) an inability to deal with complexity, (4) an inability to maintain relationships with a network of contacts, (5) a failure to delegate, (6) the allowance of emotions to cloud their judgement, (7) an inability to build a team, (8) a slowness in learning, and (9) an overriding personality defect. Bentz’s research would spark streams of research on leader derailment that would ultimately span four decades.

Two research ‘schools’ of thought on leader derailment have emerged—the first focusing on leader behaviors or behavioral derailers and the second examining the underlying interpersonal schemas and personality attributes behind these behaviors. The ‘behavioral school’ of derailment has a long history and is primarily associated with researchers at the Center for Creative Leadership (CCL). Building upon Bentz’s work, Morgan McCall and Michael Lombardo (1983) [24] studied derailed leaders in executive positions, comparing them with groups of successful executives. Their research discovered that the successful and derailed executives shared a surprising set of common factors. Individuals in both groups (1) were identified early-on as having potential, (2) were described as bright, (3) possessed outstanding records of achievement, (4) were seen as ambitious, (5) were willing to sacrifice, and (6) possessed few observable faults. That said, ten reasons would later set apart the executives who derailed in their careers. These included (1) specific business problems; (2) personal insensitivity; (3) cold, aloof, arrogant behavior; (4) betrayal of trust; (5) overmanaging; (6) being overly ambitious; (7) failing to staff effectively; (8) an inability to think strategically; (9) an inability to adapt to a boss with a different style; and (10) being overly dependent on a mentor or advocate. In addition, McCall and Lombardo (1983) [24] noted that as job demands changed over a career, some early strengths transformed themselves into weaknesses, and certain early weaknesses—when unaddressed—carried greater consequences at more senior leadership levels.

Building upon McCall and Lombardo’s work, other researchers [25,26,27,28,29,30,31] would examine the topic of derailment for another two decades. While their research samples employed a diverse range of populations and utilized diverse methods to examine leader derailment, several consistent and generalizable conclusions emerged. Most of the derailers identified in early research reappeared in later studies. For example, the findings from a methodologically rigorous study conducted by Rasch, Shen, Davies and Bono (2008) [32] highlight the degree of consistency across three decades of prior research. Their taxonomy of derailers included (1) persistent ‘people problems’; (2) poor emotional control; (3) being over-controlling; (4) poor task performance; (5) poor planning, organization, and/or communication; (6) rumor-mongering and inappropriate use of information; (7) procrastination; (8) failure to consider human needs; and (9) failure to manage and nurture talent. The category of behavior that had the most impact on staff morale was a “failure to consider human needs.” Consistent with earlier studies, they discovered that the frequency of this behavior increased with organizational status; the more senior the manager, the more pronounced and abusive the behavior. As Kaiser and Hogan (2007) [33] suggest, status in organizations allows people more discretion in their actions; with greater discretion, the derailing attributes have greater freedom to express themselves.

A ‘derailment syndrome’ also appears as a consistent pattern in the CCL streams of research. Weaknesses tolerated early in a career eventually become a serious matter, as an individual is promoted to more senior levels. In other words, supervisors downplay, or overlook, or fail to appreciate potential derailers in the individual’s early jobs by focusing on the individual’s strengths. This encourages the promoted individual to assume incorrectly that their weaknesses are not likely to become serious liabilities as the individual progresses upward in the hierarchy. Promotions and performance reviews also de-sensitize managers to the risks of overusing their strengths—another source of derailing behavior. As a result, promotions encourage overconfidence, lessen the individual’s receptivity to the value of critical feedback, and reinforce a high-performer identity. Finally, those who derail often lack strong, positive relationships with colleagues. As Van Velsor and Leslie (1995) [28] observed, when relationships are strong, people will forgive mistakes. When relationships erode, tolerance disappears. Mistakes ultimately undermine support for a leader, and their influence collapses, resulting in derailment. In contrast, successful leaders understand the need to make significant shifts in their behavior as assignments and faster rates of promotion place unfamiliar and complex demands upon them. They value strong and positive working relationships and work diligently to build positive work relationships.

The second school of derailment research posits that underlying these derailing behaviors are personality disorders and unconscious drivers [13,34,35]. These disorders and drivers produce the dysfunctional styles that prevent individuals from accomplishing the leadership tasks of building teams and creating strong interpersonal relationships. One complicating factor is that these personality disorders co-exist with ambition, expertise and social skills [34]. This allows individuals to be promoted into bigger and bigger leadership assignments. In the roles ahead of them, however, these disorders become too pronounced and eventually cause the individual’s failure. Because they are self-absorbed, derailing managers are unable to accept responsibility for their maladaptive behavior. As a result, they cannot adapt their behavior within sufficient time to pre-empt their derailment [7]. Hogan and Hogan (2001) [36] identified 11 personality disorders that are predictive of leadership derailments. Employing the *Diagnostic and Statistical Manual of Mental Disorders* (*DSM—IV*), their inventory of personality disorders includes descriptive attributes, including (1) excitable, (2) skeptical, (3) cautious, (4) reserved, (5) leisurely, (6) bold, (7) mischievous, (8) colorful, (9) imaginative, (10) diligent, and (11) dutiful. Paradoxically, each disorder is actually intended to make a positive impression in the short run — which it often does [34]. Thus, it becomes difficult to initially detect their ‘dark side’ expression. For example, Hogan and Hogan (1997) [34] describe the positive manifestation of the boldness dimension as an expression of confidence and charisma. When over-used, however, boldness is experienced as arrogance and a sense of entitlement. With boldness as their disorder, leaders overestimate their competence while failing to learn from mistakes. Low scores on these derailer disorders can be equally problematic. For example, a low level of imaginativeness often indicates a lack of vision, and a lack of boldness can translate into indecisiveness. Low scores on disorders therefore can produce leader failures as we ll as the high scores can.

What are the implications of the findings of these schools of research for the field of leadership development? As mentioned earlier, there is a remarkable consistency in the attributes of leader derailers—across genders, hierarchical levels, sectors, and cultures. From the standpoint of training, the apparent universality of both derailer behaviors and derailer disorders means that educational initiatives are able to employ standardized content. In other words, ‘derailer’ frameworks (e.g., ‘anti-leadership’ competencies) would not need to be highly customized to an organization. Similarly, standardized assessment tools incorporating derailment behaviors/disorders could be universally employed to ensure that selection into leadership positions was more rigorous. These assessments would assist the recipients and their supervisors in identifying and addressing the presence of derailer disorders. The derailment pattern of weaknesses tolerated early in an individual’s career would suggest the need for more rigorous education for supervisors in how to identify derailer behaviors and how most effectively to provide near-term corrective feedback and coaching. Similarly, junior managers require training in how to seek out and receive constructive feedback about their potential derailers. These would be baseline interventions if the field wished to move towards a more ‘preventative medicine’ model. That said, some would argue that more rigorous selection processes designed to weed out managers possessing leader derailers could be the most effective solution to the derailment challenge.

## 4. Selection: The Most Efficient Solution?

Rather than expending resources on leader development interventions, why not simply ‘select out’ of promotion opportunities those individuals exhibiting leader derailers? Several factors argue in favor of this option for addressing derailment. As noted earlier, the research shows that managers who exhibit derailers often have inflated self-evaluations [30,37,38]. They are described as highly narcissistic. A recent investigation of narcissism analyzing 51 studies with more than 37,000 participants across North America, New Zealand and Europe suggests narcissism is more stable than other personality constructs and declines with age only very gradually [39]. Those possessing derailers associated with narcissism are therefore likely to be resistant to critical feedback, learning and coaching. In other words, development investments may have little or no impact on them. Harnessing more rigorous approaches to selection in order to ‘weed out’ these individuals from leadership roles is a viable solution. If this is the case, then organizations need to devote greater attention to increasing the rigor with which they assess candidates under consideration for leadership roles. Here assessment tools such as the *Hogan Leadership Results* and the Center for Creative Leadership’s *Benchmarks* may be useful assessments when supplemented with other data sources such as performance reviews [40].

Beyond assessment tools, one promising example of a multi-dimensional selection process comes from the U.S. Army [41]. In response to two surveys, the Army reinvented its selection process for first-level executive leaders—the battalion commanders. The first survey, of 22,000 soldiers, showed that twenty percent of the respondents said they were serving under a toxic leader. The second survey indicated that less than 50% of Army majors believed that the armed service promoted its best leaders. Given the surprisingly negative results, the Army decided to reinvent its selection process for one class of senior leaders. Its aim was to intentionally weed out candidates possessing toxic or derailer attributes and to select ‘in’ those with constructive leadership skills.

The cornerstone intervention is an intensive four-day evaluation program in which promotion candidates undergo cognitive and strategic talent assessments, psychometric tests, psychological interviews, writing-skills and argumentative-essay examinations and a physical fitness test [41]. Employing a team-based outdoor obstacle course, candidates are also evaluated on their leadership and decision-making skills. In addition, peer and subordinate evaluations are carefully reviewed for any evidence of potential derailment disorders and toxic traits. The four-day assessment ends with 30 minute interviews by an evaluation panel, the members of which make the final decision on whether a candidate should be promoted. The Army requires that only the leadership levels above battalion commanders can make a final selection decision. Given this requirement, the panel consists of three one- or two-star generals and two senior colonels who have been identified as effective battalion-level commanders. In addition, there are three non-voting members of the panel—a command sergeant major with extensive experience advising battalion commanders, a senior operational psychologist and the panel moderator. To pre-empt selection errors, each panelist receives in-depth training on the attributional errors that commonly occur during job interviews. Before they begin each daily evaluations, the panelists receive an anti-bias refresher. The panel’s senior psychologists supervise junior colleagues who conduct one-on-one in-depth interviews with the candidates. These interview results are then presented in a standardized format by the senior psychologists who synthesize each candidate’s overall assessments into a summary of strengths, weaknesses and disorders. They also propose follow-up questions for the panelists to raise in the final candidate interviews. This multi-data source, multi-rater approach ensures more diverse perspectives on each candidate [41]. Panelists are not allowed to evaluate candidates if they have any prior knowledge of the individual. Even the senior psychologists are kept at arm’s length from each candidate. This ensures that the evaluators have no preconceived notions of the candidates they will be interviewing. While aspects of the evaluation, such as the obstacle course and physical fitness tests, might be of less relevance for non-military organizations, the variety of data collected and the methods of analysis could be a template for many organizations. The rigor of the process ensures a high probability that derailer behaviors and psychological disorders will be identified before the actual promotion decision is taken.

Are there drawbacks to this ‘selection-centric’ solution of weeding out individuals with leader derailers? There are several arguments against selection as a standalone intervention. For example, Hogan and Kaiser (2005) [18] attribute the majority of executive derailments *to flawed selection*. They observe that the most common selection approach is to employ interviews. Yet leader disorder attributes can actually create an initial favorable opinion—as in the example of boldness cited earlier, which creates the positive impression of confidence. As a result, interviews can be one of the weakest means used to identify those with derailment potential. For this reason, the U.S. Army has invested heavily in training its assessors in the attribution errors that occur in interviews. In addition, former subordinates’ perspectives on a candidate are rarely consulted, especially in cases involving outside candidates. Assessment tools that focus on derailer behaviors and disorders are similarly rarely employed. With a sense of irony, Hogan, Hogan and Kaiser (2010) [7] observe that many executive leaders are selected and hired for the very characteristics that will eventually cause them to derail. Hogan et. al. (2010) [7] also raise the issue of whether mitigating derailment through selection is realistic, given the small pool of effective leaders in most organizations. With the percentage of ineffective managers in their potential base reaching as high as 50%, organizations have to survive and adapt with the managers they have. There are otherwise too few viable candidates for promotion. Leader development may be the only viable long-term solution for ensuring a large-enough pool of leadership talent.

## 5. Pre-Empting Leadership Derailment Through Leadership Development: Where to Focus?

A core challenge described in the research literature on derailment is that leaders tend to derail at more senior levels of their organizations. For this reason, the response is often to deploy executive coaches to ‘turn around’ the failing leader. Yet we have no empirical evidence confirming the efficacy of ‘last ditch’ coaching attempts to salvage the career of a derailing leader. Such efforts may bear little fruit in terms of turning around the actual behavior of the leader. Why not focus greater attention on interventions at junior levels of organizations to inoculate against derailer behaviors and disorders before they become serious liabilities? What follows are a set of recommendations for developmental interventions used to not only build awareness of the derailers and disorders but to mitigate their expressions. Five interventions will be described which ideally would be employed as a comprehensive suite of initiatives. These are grounded in insights from the research literature on leadership development. For example, the literature suggests that certain methods are more effective in helping managers to develop both the intrapersonal and interpersonal skills needed to overcome derailment [9,10,42]. Knowing derailer behavior is a necessary first step, and hence the first intervention to be proposed. Among the foundational requirements are comprehensive and competent assessments supported by effective coaching and training (the second, third and fourth interventions proposed below). When successful, these interventions provide a realistic portrait for the individual of their potential derailers and the conditions that promote them. Examining the individual’s implicit theories of social interaction with their coach reveals the shortcomings of these theories within the current context. In turn, individuals will learn how their self-defeating schemas, once adaptive in earlier contexts, are now no longer viable and have become self-destructive [7]. This process, however, requires a specialized type of coaching, and for this reason we propose a fourth intervention of training for coaches. Since transitions are a common contributor to derailment, our fifth and final intervention is proposed. While not as pre-emptive as the first four, it nonetheless may provide managers with an ‘early warning’ of an impending derailment.

### 5.1. Publicizing and Socializing Frameworks of Leader Derailers

Competency models have long been used as an essential component in leadership development—especially for training programs. They are a vehicle used to publicize and socialize those behaviors considered the essential leadership behaviors within an organization. They also commonly become a foundational element in designing training content. Why not publicize the non-desired or ‘wrong stuff’ leadership behaviors using ‘anti-competency’ formats? Why not design training content around these derailer ‘anti-competencies’ while highlighting the shorter- and longer-term negative outcomes? Given the remarkable consistency in derailers described by research across varied contexts, organizations could compile ‘leader derailer’ frameworks. Sampling three to five years’ worth of known leader derailments, an organization could select the more representative derailers. These would in turn be widely publicized through training and by incorporating them into performance reviews.

The author is aware of only one organization encountered in his career that has formulated and deployed such a leader derailer framework (spelling out the behaviors that have led to derailment across all of its managerial ranks). This organization’s ‘anti-leadership competency’ framework was incorporated into leadership training programs in the form of cautionary case-studies of actual—though disguised—leaders who had derailed. Supervisory training was offered—along with assessment tools—in how to identify and coach individuals with derailers. Anecdotal evidence solicited from the organization’s CEO, CHRO and other executives suggest that leader derailment did indeed decline. They attributed the decline to a heightened sensitivity to derailer attributes which led to earlier identification and remediation through coaching and training.

### 5.2. Incorporating Derailers into 360-Feedback Leadership Surveys

One common theme in the derailment literature is a lack of self-awareness [7]. Managers who are prone to derailment view themselves more positively than others do and often externalize their personal problems. Examining multi-rater assessments, Eichinger and Lombardo (2003) [30] found that managers whose self-ratings were noticeably higher than those provided by their colleagues were more likely to fail. To complicate matters, those who will derail—like their successful counterparts—are ambitious, technically skilled, and possess strong track records. As a result, it can be difficult to discern the derailing manager from the successful one based on performance outcomes. For these reasons, the majority of performance reviews by superiors may not provide sufficient and compelling feedback on potential derailer behaviors [7].

With their tendency to focus only on ‘bright side’ characteristics of [43], current 360-degree feedback tools fall short in helping to identify an individual’s derailers. As Lombardo, Ruderman and McCauley (1988) [27] have shown, leadership failure is more often an outcome of having the ‘wrong stuff’ rather than lacking the ‘right stuff’. Moreover, the research shows that overused strengths can similarly be associated with [44]. Yet as Hogan, Hogan and Kaiser (2010) [7] point out, high ratings on the typical 360-feedback tool are generally seen as positive indicators of leadership. A high rating on a strength could be mis-interpreted as a positive indicator when in reality it is an indicator of over-use and suggests derailment potential.

To address these shortcomings with 360-degree leadership feedback tools, one solution would be to incorporate derailer attributes [7,45]. With overused strengths, feedback tools could be designed to bring attention to the links between the overuse of strengths and performance problems. The author is aware of one 360-feedback tool in which respondents—in a qualitative comments section—are able to share their observations of how a manager’s excessive use of a strength is creating performance liabilities. Another option is to pair 360 assessments with psychometric tests designed for workplaces such as the *Hogan Development Survey* [35] and the *Questionnaire of Personality Styles* [46], which focus primarily on derailers.

Surprisingly, there has been little research on, or experimentation with, 360-degree assessments that incorporate leader disorders/derailers [47]. The limited evidence suggests the following. When the self-ratings and other ratings are in agreement, with high ratings as to the presence of derailer behaviors, the individual’s leadership effectiveness is indeed perceived to be low. When the individual being assessed underrates their derailment factors in comparison to their raters’ perceptions, the individual tends to have low leadership effectiveness ratings. As might be expected, low ratings of the presence of derailers by both the manager and their raters are correlated with higher levels of leadership effectiveness. The research by Tang, Dai and DeMeuse (2013) [47] suggests that 360 surveys incorporating derailer behaviors could indeed be a helpful and potentially pre-emptive source of feedback. One constraint, however, may be the visibility of derailment factors at lower levels. For example, Tang, Dai and DeMeuse (2013) [47] did find that higher-level managers were rated higher on derailment factors than were lower-level managers.

In conclusion, feedback is an essential intervention used to address derailment potential. It appears to be underleveraged—in assessments and in performance and developmental reviews—in building greater self-awareness and promoting behavioral change. The research suggests that this tool can often lead to positive change, especially under certain conditions. As summarized by Hogan, Hogan and Kaiser (2010) [7], these conditions ideally include situations (1) when feedback is intended for development versus administrative reviews, (2) in which coaches are available to help managers review and set improvement goals, (3) in which managers share their development goals with colleagues and engage them in action planning, and (4) the feedback is for the lowest-rated managers.

### 5.3. Targeted Training on Leader Derailment

It is rare to hear of leadership training programs with a focus on derailment. This is likely one outcome of the positivist bent of the leadership development field. It may also be a recognition that resolving derailers in a training program is an unrealistic expectation. After all, many of the derailer behaviors have their roots in personality disorders. Training programs would be hard-pressed to adequately tackle the emotional and motivational causes underlying many derailers.

The present author is not so pessimistic about the potential of training, especially with more junior audiences and with the frame of building awareness. He is familiar with one such program for high-potential leadership talent. The program’s training content explored the ways in which managers in the organization commonly derailed in their careers. Educational content covered relevant lessons from the research on leader derailment while case studies explored the overuse of strengths, derailer disorders, and the cycle of derailment. Each participant received feedback on their own potential to derail from the *Hogan Leadership Forecast* assessment. In addition, participants had three coaching sessions to devise action plans to address their potential derailers and/or leadership skill gaps as well as develop constructive leadership skills. To build the case for the program, the organization’s CEO opened by sharing his decision to terminate the firm’s chief engineer (whose leadership role oversaw several thousand engineers) due to his derailer behaviors. The CEO described reviewing twenty-four years of the chief engineer’s performance evaluations to make his termination decision. The behaviors that led to this executive’s derailment were found in every single performance review since the start of his career. Behaviorally, he was distant, exhibited poor emotional control and failed to nurture and mentor talent. The story’s parallel to the research literature was striking—individuals promoted with their weaknesses until these weaknesses become too pronounced and could no longer be tolerated. This powerful story left an indelible mark on the program participants.

There may indeed be a place for training and its capacity to build a persuasive case to address one’s derailers early in a career. At a minimum, training can be employed to (1) build awareness of common derailers and how leader derailments typically unfold, (2) help participants identify and begin to address their own potential derailers, (3) teach individuals how to proactively solicit constructive feedback and act on it, (4) socialize expectations that derailer behavior will not be tolerated by the organization, and (5) simultaneously develop constructive leadership skills. As Day (2024) [9] has noted, one challenge facing training approaches is their short-term effects. If individuals do not practice consistently what is learned in the training experience, then ‘decay’ becomes the more likely outcome. For reason, follow up coaching by supervisors or professional coaches is essential requirement. 

### 5.4. Developing Coaching Skills Specifically for Addressing Derailers

As noted earlier, coaching is often seen as the ‘fix-all’ for derailing leaders. Indeed, coaching is more effective than training because it can be customized to the individual and occurs over extended time periods. That said, the professional attributes required in these coaching engagements are complex. Paradoxically, many programs that teach coaching skills do not necessarily focus on addressing derailing leaders in their curricula. Since these coaching engagements must address underlying personality disorders, they necessitate training in psychotherapeutic techniques. As Sargent (2011) [48] notes, the requisite coaching attributes include: (1) discerning the client’s degree of readiness for change; (2) possessing a portfolio of experiential and behavioral strategies and interventions; (3) understanding the client’s emotional state and when it is appropriate to apply each strategy and intervention; (4) staging experiential interventions to raise awareness, build resilience and stimulate the desire to experiment and change; and (5) introducing behavior-based interventions to encourage clients to take practical steps facilitating change. Given this list, it is not surprising that, just two decades ago, the coaches employed for derailing executives were primarily psychologists and psychiatrists. The number of coaches who are highly skilled across this suite of attributes is likely to be small in number. As Kirschner and Zamora (2022) [49] suggest, it is highly recommended that coaches who undertake these assignments themselves seek regular supervision from peer coaches to navigate their own psychodynamics (transference/countertransference) relative to their clients and to bolster their senses of self-efficacy and resilience in the face of defensive client behavior. Kets de Vries (2006) [36] explains the psychoanalytic skills required to coach those most prone to leader dysfunction and derailment. 

Hogan, Hogan and Kaiser (2010) [7] highlight what these coaches—beyond Sargent’s list of required skills—must accomplish with derailing leaders as their clients. First, they must assist each client in surfacing the individual’s implicit theories of social interaction and assumptions about motivation. In the process, they have to build an awareness of the individual’s faulty assumptions, self-defeating schemas and emotional triggers/hot buttons and how these currently manifest themselves in highly dysfunctional ways. The coach must show how these self-defeating schemas are no longer adaptive—even though they may have been successful in earlier contexts. They then reprogram the client’s faulty schemas by supplanting the counterproductive behavior with new constructive leader behaviors that are reinforced through recognition and positive performance outcomes. Eventually the new behaviors become self-sustaining. As one might imagine, this is a time-consuming process often characterized by as many setbacks as times of forward progress. Behavior change in a derailing leader is further complicated since many are unwilling to take responsibility for their failings and are resistant to coaching and feedback. We can conclude that many coaches are not well prepared to successfully coach derailing leaders. Therefore, careful selection of coaches is a necessity. The skills required also suggest that most supervisors of derailing individuals will not possess the requisite coaching skills themselves.

It has been suggested that peer coaching could play a role in building accountability and support for behavioral change with derailing leaders. It is certainly a more cost-effective solution than utilizing professional coaches. But this option is not likely to be a viable for several reasons. Peers often have to maintain a façade of likeability to ensure positive working relationships. They may lack the confidence required to constructively confront a colleague who is derailing—fearing resentment or resistance if the derailing peer feels judged or threatened by them. Moreover, peers are likely to lack the sophisticated coaching skillsets needed to successfully support the complicated behavior-change process.

### 5.5. Formalize Onboarding Interventions for Job Transitions

One consistent trigger for derailments is the transition into a new role or to work under a new supervisor [50]. This is particularly true with transitions to senior leadership roles [51]. With these promotions come far greater visibility, more scrutiny, organizational politics which are more complex, and often-ambiguous performance expectations [52,53]. For example, newly promoted executives must address three dilemmas [54]. First, in a relatively short period of time, they need to gain mastery over a complex and demanding role. The learning demands are dramatically elevated and may be the most pronounced ones in a manager’s career. Second, expectations are high that the incoming executive already has the capability to lead in the new role. In other words, they require little or no ‘honeymoon’. As a result, developmental feedback and support are rare for those in the upper echelons of organizations. These two challenges lead to the third dilemma. The probability of a new executive’s derailment is high. Complex new role demands combined with a lack of developmental support can produce a ‘perfect storm’—the failure of the new leader.

Despite these demands, organizations rarely prepare or effectively ‘onboard’ leaders into senior roles. As noted in the research, weaknesses that mattered less in junior roles or strengths that were highly valued in a prior role now have the potential to become derailers. Yet if organizations are to mitigate derailments at their most senior levels, they must make serious investments in onboarding programs—utilizing a full range of interventions from assessments, to coaching, to feedback, to training. One in-depth longitudinal case study [54] provides baseline insights into the program-design features that make for a successful onboarding experience. The first is to recognize that onboarding occurs over a time frame from twelve to eighteen months on the job—not simply the first 90 days. Onboarding process therefore must be supported by *multiple interventions* instead of a single ‘welcome on board’ event at the moment of entry. To ensure continuous feedback, developmental interventions need to be spaced at *intervals* over the executive’s first year to 18 months rather than within the first few months in the job. The fullest possible spectrum of stakeholders should be involved in the selection, entry and onboarding. All too often, the new executive’s supervisor is the only individual engaged in the onboarding process. Utilizing coaches and feedback tools, stakeholders need to provide ongoing, candid feedback to the new leader. Coaches, for example, may be able to solicit richer, more accurate feedback from the leader’s stakeholders when it is provided without attribution to specific individuals. In any case, the new executive needs to be aware of expressions of derailer behaviors as well as behaviors that are highly effective. Finally, onboarding interventions are completely dependent upon the quality of the *interaction* between the executive and their stakeholders. A bureaucratic process will not succeed. The approach must focus on the quality of candid *dialogue* rather than documentation and formal processes. The new executive leader must receive ‘in time’ critical, candid and valid feedback so that they can constructively respond and maintain or regain their credibility. Only with rigorous designs can onboarding programs play an essential role in pre-empting some of the most visible and costly leader derailments.

## 6. Conclusions

The research on leadership derailment has a long and rich history. Given the variety of studies, there is a remarkable level of convergence in terms of our insights into the behaviors and underlying personality disorders that ultimately produce leadership failures. Yet this body of knowledge seems to remain in a parallel universe relative to the field of leadership development. Given the remarkable costs associated with derailments, we need to integrate and translate this knowledge into rigorous inventions that have the potential to mitigate derailments. The assumption that instilling constructive leadership competencies is sufficient to address derailment is misplaced. The ‘wrong stuff’ attributes are indeed distinct from the ‘right stuff’ ones—as evidenced by decades of leadership development initiatives and expenditures in the billions of dollars that have yet to resolve the widespread problem of leadership derailments. We need a new paradigm within leadership development—one that emphasizes the need to (1) directly address derailers, doing so (2) with a richer set of interventions and (3) at earlier stages of a manager’s career, and (4) to encourage scholars and practitioners in the leadership development field to invest in greater research. The long-standing reliance on ‘last minute’ coaching as the solution is costly in so many ways. This article and its recommendations serve as a call to action.

In terms of the limitations of the conclusions in this article, it is important to recognize that this is a conceptual paper. It needs to be backed up by research examining the efficacy of the five interventions which are proposed. For example, we know little about the coaching skills required to assist managers who are derailing. The work by Kets de Vries (2006) [35] offers insights into the clinical psychotherapeutic requirements that are likely to be foundational. We do not know whether it is feasible to discern derailers early in a manager’s career. The assumption that early career exposure to the personality attributes and behaviors associated with derailment is sufficient to pre-empt leader derailments itself needs to be rigorously tested. Research investigations focusing on the following topics would advance our understanding of the topics in this article: (1) the utility of training and assessments as tools used to build awareness and ultimately pre-empt leader failures, (2) the coaching skills required to address derailing leaders and whether these are distinct from what is currently taught, (3) the development interventions for leaders transitioning into new roles which can be used to identify and address the impacts of their derailing behaviors, and (4) the effectiveness of training supervisors in the capacity to detect derailing personality attributes and to provide effective feedback. It is clear that there are significant opportunities for future research that could have a profound impact on pre-empting leadership derailments.

## Data Availability

Data are contained within the article.
